# Tipping the scales of understanding: An engineering approach to design and implement whole-body cardiac electrophysiology experimental models

**DOI:** 10.3389/fphys.2023.1100471

**Published:** 2023-01-19

**Authors:** Brian Zenger, Jake A. Bergquist, Anna Busatto, Wilson W. Good, Lindsay C. Rupp, Vikas Sharma, Rob S. MacLeod

**Affiliations:** ^1^ Scientific Computing and Imaging Institute, University of Utah, Salt Lake City, UT, United States; ^2^ Nora Eccles Harrison Cardiovascular Research and Training Institute, The University of Utah, Salt Lake City, UT, United States; ^3^ Spencer Eccles School of Medicine, University of Utah, Salt Lake City, UT, United States; ^4^ Department of Biomedical Engineering, College of Engineering, University of Utah, Salt Lake City, UT, United States; ^5^ Acutus Medical, Carslbad, CA, United States

**Keywords:** cardiac electrophysiology, experimental models, acute myocardial ischemia (AMI), torso tank, isolated heart, electrocardiographic imaging (ECGI)

## Abstract

The study of cardiac electrophysiology is built on experimental models that span all scales, from ion channels to whole-body preparations. Novel discoveries made at each scale have contributed to our fundamental understanding of human cardiac electrophysiology, which informs clinicians as they detect, diagnose, and treat complex cardiac pathologies. This expert review describes an engineering approach to developing experimental models that is applicable across scales. The review also outlines how we applied the approach to create a set of multiscale whole-body experimental models of cardiac electrophysiology, models that are driving new insights into the response of the myocardium to acute ischemia. Specifically, we propose that researchers must address three critical requirements to develop an effective experimental model: 1) how the experimental model replicates and maintains human physiological conditions, 2) how the interventions possible with the experimental model capture human pathophysiology, and 3) what signals need to be measured, at which levels of resolution and fidelity, and what are the resulting requirements of the measurement system and the access to the organs of interest. We will discuss these requirements in the context of two examples of whole-body experimental models, a closed chest *in situ* model of cardiac ischemia and an isolated-heart, torso-tank preparation, both of which we have developed over decades and used to gather valuable insights from hundreds of experiments.

## 1 Introduction

Cardiac electrophysiology is a discipline that plays out on a wide range of scales, from ion channels and cardiac myocytes to the whole body and torso-surface recordings (Katz, 2010). Experiments that have established and validated mechanisms across these scales have advanced our understanding of cardiac function, development of pathologies, and targeted treatments (Zipes et al., 2014). At each scale, experimental models, typically based on animals, are necessary to examine electrical function in ways that go beyond what is feasible in humans. The nature of electrophysiology and the associated access to electrical parameters have both enabled and required researchers in the field to create and use models across organ and whole-body scales. These models are the closest approximations of humans and enable translations from bench-top measurements to bedside treatments (Cluitmans et al., 2018; Cuculich and Robinson, 2018; Grace et al., 2019). The models are typically based on larger animal species, such as pigs or dogs, and support a wide range of cardiac pathologies (Dosdall et al., 2013). The need for carefully designed experimental models extends well beyond electrophysiology as these models allow researchers to interrogate specific aspects of cardiac physiology, develop hypotheses, and answer important questions to improve our understanding, detection, and treatment of cardiac disease.

Historically, experimental models of whole-body cardiac electrophysiology were complex and resource intensive; in return for this investment, such models have provided valuable data to understand human electrophysiology (Spach et al., 1977; Dosdall et al., 2013; Bear et al., 2018; Zenger et al., 2021a; Zenger et al., 2021b). Whole-body experimental models have been used to examine the development (Li et al., 2001; Neuberger et al., 2005) and treatment modalities (Calzolari et al., 2017; Solimene et al., 2019; Koruth et al., 2020) for atrial fibrillation (AF), including the seminal hypothesis “AF begets AF” (Wijffels et al., 1968). Large-scale experimental models have been used to test and validate novel imaging and electrical localization techniques, including electrocardiographic imaging (ECGI) (Ramanathan et al., 2006; Bear et al., 2018; Cluitmans et al., 2018; Cuculich and Robinson, 2018; Grace et al., 2019; Bergquist et al., 2021). Furthermore, animal models have enhanced our understanding of cardiac signals that develop during acute myocardial ischemia and how these signals change in various cardiac stress circumstances (Aras et al., 2011a; Aras et al., 2016; Zenger et al., 2021a; Zenger et al., 2021b; Good et al., 2021). Animal experiments play many key roles in exploring mechanisms and often in providing behaviors and parameters included in subsequent numerical and computer modeling (Plank et al., 2008; Quinn et al., 2011; Prakosa et al., 2019). Computational models are synergistic to this process, providing quantitative evaluation of proposed mechanisms and, most recently, diagnostic prediction and therapeutic guidance. However, all computational models must be validated and in many cases, such validation is challenging or untenable in humans so that animal experiments, often with large mammals, are required. As a result, even in an age of dramatic progress in digital-twin approaches, experiments retain their essential value and best serve their purpose when coupled with simulations. Another often overlooked contribution of large animal experimental models is their use in veterinary medicine (Ranjan et al., 2014). Thus, while the use of animals in medical research also bears an ethical burden, such models have provided essential insights into both human cardiology and veterinary care.

In this article, we outline a systematic engineering approach that we have used to design and implement whole-body experimental models to answer relevant questions in human cardiac electrophysiology. The tools and framework discussed in this article are designed to support scientists and engineers developing or expanding experimental models in cardiac electropysiology and bioelectricity at organ-scale. We know of few laboratories, academic or industrial, that have focussed attention on this domain and even fewer attempts to summarize the resulting insights and general approaches to such models. This article also supports scientists with clinical or computational modeling backgrounds by providing a framework to evaluate an experimental model design. We discuss several nuances we considered in our experimental design that could apply broadly to other approaches. Specifically, we propose that researchers must address three critical questions to develop an effective experimental model in cardiac electrophysiology: 1) how does the experimental model replicate human physiology, 2) how can researchers manipulate the experimental model to faithfully replicate human pathophysiology, and 3) what signals need to be measured and how will they be recorded? We will discuss these questions in the context of two examples of whole-body experimental models, a closed chest *in situ* model of cardiac ischemia and an isolated-heart, electrolytic phantom, or torso-tank preparation. We have developed these preparations over decades from hundreds of experiments and used them to gather valuable insights. Our perspective will focus on cardiac electrophysiology, but these same principles can guide the study of any aspect of physiology and pathophysiology.

## 2 Engineering requirements of experimental models

### 2.1 Defining the project goal and generating testable hypotheses

Step zero in all experimental model designs should clearly outline the project goal and testable scientific hypotheses. Clearly outlining the scientific question and hypotheses promotes an experimental model design that will 1) focus on the relevant aspects of physiology and pathophysiology that the model must replicate, and 2) ensure that the model produces the necessary measurements to test the scientific hypothesis. The methods of establishing and curating scientific problems and testable hypotheses are outside of the scope of this article, which focuses on experimental model design after a scientific question and hypothesis has been established.

### 2.2 Similarities to human physiology

The first component to consider when exploring experimental model selection, especially in large-scale models, e.g., canine and porcine (Macfarlane et al., 2010; Dosdall et al., 2013), is the similarities to human cardiac electrophysiology, including gross cardiovascular anatomy, electrical conduction system anatomy and function, and coronary vascular anatomy. Gross cardiovascular anatomy describes the number and shape of cardiac chambers, the number of large vessels, and the overall orientation of organs within the thoracic cavity (White et al., 1992; Lelovas et al., 2014). Overall, most large mammals used for such research have similar anatomies with subtle differences in lung volume, e.g., dogs tend to have significantly larger lungs than pigs, which wrap around the anterior surface of the heart (Lelovas et al., 2014). Electrical conduction system anatomy is also a critical aspect of structure and function; it relates to the organization of the pacemaker, AV node, and most importantly, the Purkinje network. Purkinje network differences are commonplace between species, with canines having more similar Purkinje systems to humans than porcines (Verdouw et al., 1983). Specifically, pigs have Purkinje fibers that run in the mid-myocardium whereas canines have a subendocardial Purkinje network (Verdouw et al., 1983). Finally, the coronary vasculature must also be considered, especially for studies involving coronary flow or ischemia. Most large mammals generally have similar gross coronary vascular structures, including the right and left coronaries with similar branches (White et al., 1992). However, the microvascular and arteriolar structure can vary across species; specifically, canines have extensive collateral perfusion whereas porcines have minimal collateral perfusion, the latter more typical of human collaterals.

### 2.3 Replication of pathologies

A second critical consideration in selecting an experimental model is its ability to replicate the human pathophysiology of interest. For studies of electrophysiology, the emphasis is on electrical features, and the animal model must allow perturbations that are similar enough to replicate the human pathology of interest. One key feature of such perturbations is their time course; do the conditions of interest arise immediately or only after a prolonged period, and how well does the animal response match human pathology over the desired time course? Examples of acute pathologies include an acute blockage of a coronary artery or localized premature ventricular contractions. Chronic pathologies include the long-term development of scar tissue or chronic cardiac rhythm perturbations. Preparations in which animals must recover from an intervention or develop a condition over days or months demand extensive resources and management. A resulting concern is the presence of unintended consequences to the primary intervention that can lead to confounding co-morbidities, for example, a model of rapidly pacing the heart to create atrial fibrillation can also lead to congestive heart failure (Dosdall et al., 2013). A further consideration is the role of inter- and intra-animal variability in response to a specific perturbation; just as with humans, it is naïve to assume that every animal, even of the same species, will respond similarly and suitably. One solution is to normalize the experimental process to accommodate animals that are more susceptible to the interventions than others.

In models of atrial fibrillation, which require recovery periods of weeks to months, dogs and goats are the current species of choice as they replicate atrial fibrillation well and can survive for many months without unacceptable distress (Wijffels et al., 1968; Dosdall et al., 2013). Importantly, in these species, induction of chronic atrial fibrillation appears not to compromise the hemodynamic stability of the animal. Pigs, by contrast, fail to maintain stable hemodynamics and electrophysiology following the rapid pacing necessary to create atrial fibrillation and are less viable models for this pathology (Buda et al., 1985). Atrial fibrillation also represents something of a special case as it also arises naturally in animals, e.g., in horses, allowing the goals of human and veterinary medicine to overlap (Linz et al., 2020). Models of myocardial ischemia and infarction are also common, either as the primary pathology or as the precursor to subsequent arrhythmias. Given the limited collateral perfusion in the coronary vasculature of pigs, minor vascular insults can produce large areas of ischemia or infarction (White et al., 1992; Lelovas et al., 2014). Therefore, such occlusions are best limited to distal coronary branches to preserve hemodynamic stability.

### 2.4 Measurement system requirements and access

Another important consideration for all experimental models is the required (or desired) measurements and how accessible they are. Most large-scale electrophysiological experimental models include extensive instrumentation, depending on the research goals. In cardiac electrophysiology, designing and fabricating devices to record these electrical signals must be carefully balanced based on the hypothesis of interest, the ability to capture relevant parameters, the clinical parallels of such recordings, and the resulting burden on the preparation. For example, electrograms can be recorded experimentally throughout the heart from inside the heart chamber and vasculature *via* catheters, from within the cardiac tissue using implanted needle electrodes, and from the heart surface using either catheter or contact electrodes. Torso potentials typically come from the body surface but can also be measured within the chest cavity. Technical considerations for each of these sources include attachment and localization of electrodes, spatial sampling density and coverage, and signal amplitudes and frequency ranges, all of which depend on the physiology of interest. Depending on the location, myocardial signal amplitudes can vary by orders of magnitude (MacLeod et al., 2010; Pullan et al., 2010; Zenger et al., 2020a), and physiological frequencies of interest may drive sampling rates as high as 10,000 samples per second (Barr and Spach, 1977), with the incurred demands on acquisition systems, storage, and processing overhead.

Once signals and images are acquired, they must also be processed and annotated to extract the necessary metrics for each scientific question. Cardiac electrophysiology is a spatio-temporal phenomenon and the variable of interest include voltage amplitudes or interval lengths, which must also be associated with underlying, subject-specific anatomy. To take advantage of new technology that enables hundreds of independents channels of data collection, we have developed tools that can robustly and simultaneously filter, organize, annotate, and visualize signals from thousands of channels (MacLeod et al., 1992; Rodenhauser et al., 2018; Zenger et al., 2020a). Furthermore, we have developed approaches to automatically annotate these signals from template recordings (Rodenhauser et al., 2018) and to assemble the complex geometric relationships between relevant anatomy and measurement locations (Bergquist et al., 2019a). This suite of open-source tools enables rapid signal analysis that supports extensive interrogation of experimental results.

A consistent requirement in large animal studies is recovering geometric information, including anatomy and electrode locations, all assembled in the same coordinate system (Cluitmans et al., 2018; Zenger et al., 2020a; Burton, 2018; Swenson et al., 2910; Bergquist et al., 2022a). The requirements for capturing this information come from the scope and focus of the research questions and include such considerations as the amount of the heart and thorax that must be captured to create a suitable geometric model. Closely related are questions of the required spatial and even temporal accuracy, coverage, and resolution. Should the geometry include all four chambers of the heart or bones, lungs, or other organs? Should cardiac or respiratory movements be accounted for, i.e., from contraction, breathing, or exercise? The technical costs of reconstructing geometry include, beyond the obvious anatomical reconstruction, the locations of electrodes and potentially the movement of organs and sensors, which are typically captured through some combination of medical imaging and physical digitizing (Durrer et al., 1970; Taccardi et al., 2008; Zenger et al., 2020a). Suitable reconstruction also requires significant post-processing to align and deform the elements based on registration across imaging modalities (Bergquist et al., 2019a; Zenger et al., 2020a).

As in all such experimental models, it is necessary to balance levels of access or invasiveness against preparation stability and physiological compromises. All scientists would appreciate an infinitely instrumented experimental model that can measure all important and potentially important signals of interest; however, such conditions are neither feasible nor practical. Recordings of both signals and anatomy must be limited to the minimum necessary to answer specific scientific questions, while ensuring realistic and stable physiological conditions. In general, measurements of extracellular bioelectric potentials are among the easiest to implement as they achieve the highest coverage and resolution under the least invasive conditions. Mechanical function is much more challenging to capture, often feasible only through indirect or integrative measures, e.g., blood pressures within chambers or vessels or through imaging modalities that capture sequences of images, e.g., three-dimensional ultrasound or four-dimensional MRI (Pahlm and Wagner, 2011). For all types of measurement, it is necessary to anticipate the presence of sensors altering function, e.g., needle electrodes that pierce the tissues or sensors that require surgical implantation, in both cases causing tissue damage or compromised function. There may even be interference among sensor types, e.g., electrodes may impede or distort image quality, or catheters may compete for limited vascular space. Finally, each animal will require some level of sedation or anesthesia, which can reduce or eliminate normal physiological responses, with deeper anesthesia needed with each degree of invasiveness.

## 3 Example: Closed chest *in situ* model of cardiac ischemia

A multiyear focus of our development and use of large-animal experimental models has been motivated by the study of acute myocardial ischemia (Shome et al., 2004; Shome et al., 2007; Aras et al., 2014; Aras et al., 2016; Burton et al., 2018a; Bergquist et al., 2019b; Zenger et al., 2020a; Zenger et al., 2021a; Bergquist et al., 2021; Zenger et al., 2021b). Chest pain, presumed to be cardiac ischemia on presentation, is the number one reason patients present to the emergency department in the United States (Bhuiya et al., 2010; Safdar et al., 2018). Furthermore, incorrect detection and diagnosis can lead to treatment delays associated with increased morbidity and mortality (Noel et al., 1092). We have designed a series of experimental models to examine components of acute myocardial ischemia, including its dynamic formation within the cardiac tissue. Here we apply the design approach described above to one such model with which we studied acute ischemia by capturing bioelectric potentials within the heart and simultaneously over the epicardial and body surfaces under conditions of a closed chest (Zenger et al., 2020a; Zenger et al., 2021a; Zenger et al., 2021b).

### 3.1 Anatomy and physiology considerations

To achieve the necessary coverage and density of bioelectrical measurements under conditions that replicate the human condition, only large-mammal models came into question for these studies. Porcine and canine species are the most suitable for such studies, and both have a long history and experience base within the field; however, we also considered anatomical and physiological differences between the species when designing the experiments. In both pigs and dogs, the heart is situated within the chest cavity and surrounded by a pericardial sack and lungs. However, there are subtle differences in the heart orientation and lung position. In both species, the apical-basal axis is oblique, almost perpendicular, to the spine when the animal is standing, whereas, in humans, this axis is parallel to the spine. There are relevant gross anatomical differences between the animal species as well, e.g., the lungs in canines are much larger than in pigs and expand around the anterior heart surface during inspiration. When in the supine position typical for experiments, the distance from the heart to the torso surface also differs slightly between canines (larger) and porcines (smaller). Furthermore, skin thickness in pigs and excess hair in dogs can impede torso surface recordings, resulting in reduced ECG amplitude and distortions in signal morphology.

The heart shape and sizes are roughly the same across canine and porcine subjects, with four heart chambers of two atria and two ventricles. Furthermore, the systemic vasculature connections (i.e., aorta, vena cava, *etc.*) are similar to humans in both species. Finally, the gross architecture of coronary circulation is similar in both species even though there are differences in the microvasculature and collateral branches (White et al., 1992; Lelovas et al., 2014). Specifically, canines have extensive, rapidly recruitable collateral vessels that can maintain cardiac perfusion if a large vessel in one branch is damaged or occluded (White et al., 1992). In pigs, similar to humans, fewer collateral vessels can be recruited quickly, with most regions of the heart perfused by one primary vessel (White et al., 1992; Lelovas et al., 2014).

The electrophysiology of the heart is similar on a cellular level among pigs, dogs, and humans and recent comparative studies even propose a scheme to translate relevant electrophysiological parameters across mammalian species (Morotti et al., 2021). At the level of the conduction system, however, the differences among canine, porcine, and humans become relevant to the study of ischemia and arrhythmias. All species have SA nodes, AV nodes, and Purkinje fibers; however, the canine Purkinje network is located within the subendocardium, similar to humans (Verdouw et al., 1983). This structure causes electrical activation in the ventricles to originate in the endocardium and progress toward the epicardium (Durrer et al., 1954; Durrer et al., 1970). Pigs, in contrast, have a Purkinje network that runs through the mid-myocardium so that electrical activation primarily originates in the mid wall and progresses in opposing directions to the ventricular epicardium and the endocardium (Verdouw et al., 1983). Such differences affect the details of the spread of activation but appear to have little impact on the basics features of the ECG, e.g., the dipole vector and gross appearance of QRS morphology (Zenger et al., 2020a). ECG intervals, including the PR, QRS, and QT intervals, are similar between dogs, pigs, and humans when scaled for heart rate (Zenger et al., 2020a). There are also interspecies differences in the measured repolarization gradient (Burger, 1957), as one would expect given the difference in the Purkinje network described above (Verdouw et al., 1983). Pigs tend to have early mid-myocardial repolarization compared to dogs, which show early repolarization in the subepicardium.

The overall cardiovascular physiology also reveals modest differences among these candidate large-mammal species. Resting blood pressure and heart rate ranges are similar in dogs and pigs compared to humans (Zenger et al., 2020a), whereas canines have a higher upper limit of heart rate resulting from evolutionary advantages related to their extensive physical activity (Macfarlane et al., 2010; Dosdall et al., 2013). Although only incompletely studied, the regulatory mechanisms that control cardiovascular function, including local vascular smooth muscle and central homeostatic control, are generally similar across large mammals. The importance of these differences depends greatly on the specific goals of the study, which in our case have been electrophysiological in nature and acute in time course (1–20 min).

### 3.2 Pathophysiology

Our next step in this experimental model was to establish a means of replicating the human response to acute ischemia in a way that allowed us to control the extent and time course of the ischemic stress to mimic clinical exercise or pharmacological stress tests (Cox et al., 1973; Downar et al., 1977; Janse et al., 1980; Janse and Kleber, 1981; Janse et al., 1998; Safdar et al., 2018). To simulate this most common anginal chest pain scenario, we reduced regional coronary blood flow and increased myocardial activity (Downar et al., 1977; Janse et al., 1980; Safdar et al., 2018). Whereas many previous studies have replicated the scenario of a myocardial infarct, typically by ligating a coronary vessel completely, (Holland and Arnsdorf, 1977; Holland and Brooks, 1977; Kleber et al., 1978; Cinca et al., 1980; Janse et al., 1980; Chang et al., 1989; MacLeod et al., 1995a; MacLeod et al., 1997), we sought a more controlled and nuanced approach that also created ischemia typical of stable transient angina (Penny, 1984; Shome et al., 2007; Aras et al., 2009; Aras et al., 2011a; Aras et al., 2016; Zenger et al., 2020a).

**FIGURE 1:**
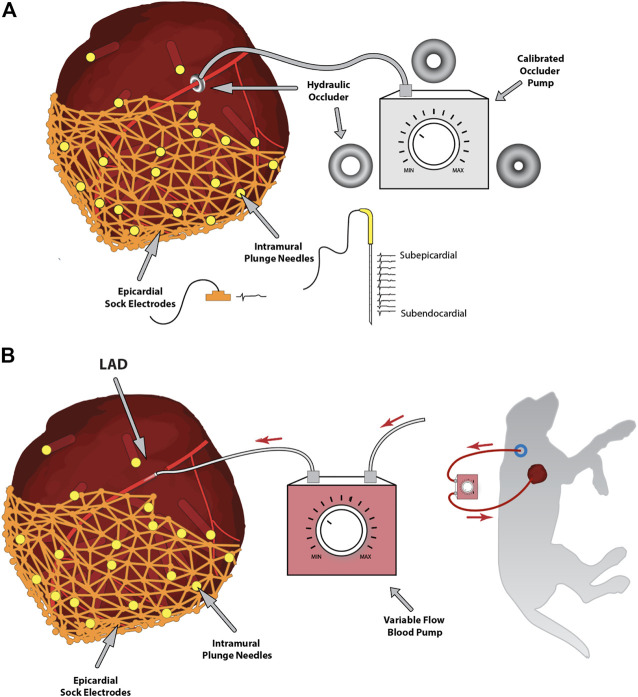
Experimental approaches to controlling coronary blood flow. Panel **(A)** shows the hydraulic occlusion device placed around the left anterior descending coronary artery and controlled *via* an external calibrated hydraulic pump. Also, note the intramural and epicardial electrical recording arrays, including samples of recorded signals. Panel **(B)** shows an alternative approach of a controlled-flow blood pump connecting a carotid artery to the left anterior descending coronary artery to control blood flow downstream of the cannulation.

To achieve this state of controlled, reduced coronary flow, we have used two approaches (Figure 1). The first was a controlled-flow pump attached to a cannulated coronary artery (Aras et al., 2016). Figure 1B shows the resulting configuration, in which blood from a carotid artery flowed through the pump to the cannulated left anterior descending coronary artery (LAD). The major advantage of this approach was precise control of blood flow into the region of the heart below the cannulation. Disadvantages included the unphysiologic, continuous flow from the pump, which does not represent the pulsatility of coronary flow, and the challenge of establishing absolute flow rates under control conditions, rates that will vary from preparation to preparation. (Aras et al., 2016).

A second approach is shown in Figure 1A, where we surgically placed a variable hydraulic occlusion device around a proximal segment of the LAD (Aras et al., 2016). The inner cuff diameter could be increased or decreased hydraulically from outside the body, adjusting the flow from fully open to wholly occluded in a calibrated manner. This approach maintained the pulsatile nature of bloodflow and adjusted automatically to baseline flow rates, mimicking the pathophysiology of reduced myocardial supply, which often occurs secondary to a blockage or coronary spasm. Furthermore, this device could be controlled externally, even through a reclosed chest, by means of a hydraulic line, and quickly inflated or deflated during experimental interventions (Aras et al., 2016; Zenger et al., 2020a).

Episodes of acute myocardial ischemia also often involve increased myocardial demand, e.g., from exercise or other physiological stress (Bhuiya et al., 2010; Safdar et al., 2018). To mimic this variable demand from exercise, we implemented both electrical and pharmacological stimulation (Heller et al., 1984; Mannering et al., 1988; Marangelli et al., 1994; Schröder et al., 1996) through right-atrial pacing and dobutamine, respectively (Zenger et al., 2021a). These protocols allowed us to collect electrograms during variable demand levels from the same subjects to examine acute myocardial ischemia and compare the results to observations in humans.

### 3.3 Measurement system requirements and access

To capture the spatiotemporal dynamics of ischemia comprehensively required a range of bioelectric potential measurements that included both surface and volumetric sensors. The results were two types of data: the signals themselves, captured continuously and at suitable sample rates, and the locations of the sensors relative to the cardiac anatomy, a much more static set of measurements that presented their own challenges. The result, based on decades of experience and development in our laboratory (Macleod et al., 1896; Franzone et al., 1978; Taccardi et al., 1994; Khoury et al., 1995; Fuller et al., 2000; Taccardi et al., 2005; Taccardi et al., 2008; Aras, 2015; Aras et al., 2016) and from others (Rogers et al., 2002; Bear et al., 2015; Trew et al., 2019), was a collection of different types of high-resolution recording arrays, including intramural multielectrode needles, epicardial socks, and torso surface strips (Zenger et al., 2020a; Zenger et al., 2020b).

**FIGURE 2:**
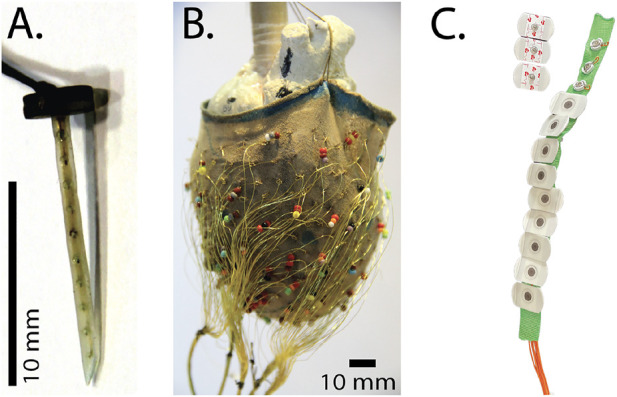
Electrical recording arrays used to measure cardiac bioelectric potentials in the experimental models. **(A)** A plunge needle electrode **(B)** a 247-lead electrode sock suspended over a plaster model during fabrication, and **(C)** A strip of torso electrodes containing 12 disposable electrodes.

We have developed three generations of intramural plunge needle arrays, all constructed by hand, the most recent of which used high-resolution 3D-printing techniques as shown in Figure 2 (Zenger et al., 2020a). Each intramural needle holds 10 individual electrodes created from the electroplated (with Ag/AgCl) tips of silver wires and evenly distributed down the shaft at spacings that reflect the thickness of the right and left ventricles. For the ischemia experiments, these needles were inserted into the anterior volume of the ventricular myocardium to sample, intramurally, the regions up and downstream of the occlusion. Key desirable features of these needles include insulation between individual wires, flexible shafts to limit the effects on contraction, and a small diameter to reduce the injury induced by placing the electrodes. To enable re-closing the chest, we also developed low-profile heads for the needles that minimized bending of the wires while limiting their protruding outside the heart.

Flexible sock arrays provided comprehensive coverage of the ventricular epicardium and were constructed using a nylon stocking with 240–490 hand-stitched and approximately evenly spaced electrodes made of silver wire with the ends exposed to form the contact electrodes (Figure 2) (Aras et al., 2016; Zenger et al., 2020a). The stocking provided stretch and compression when placed on the epicardial surface to ensure electrode contact throughout the cardiac movement. To fabricate these arrays, we stretched the toe area of pantyhose legs over plaster molds of hearts covered with markings for the desired electrode locations, and then tied the stripped ends of insulated silver wire (diameter 77–127 *μ*m) around the strands of nylon to anchor the electrodes.

Finally, we developed linear strips of torso electrodes based on the CVRTI body-surface-mapping system (Abildskov et al., 1976; Lux et al., 1979). Each strip contained 4–12 electrodes with spacing selected based on animal size and desired coverage. Initial designs used customized electrode pellets from sintered Ag/AgCL, whereas more recent implementations have used standard, disposable electrodes clipped to customized back strips and attached to the shaved skin *via* disposable adhesive electrodes (Figure 2) (Zenger et al., 2020a; Zenger et al., 2021a; Zenger et al., 2021b).

**FIGURE 3:**
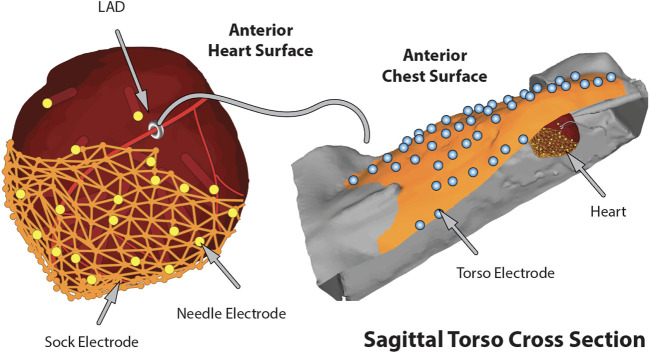
The complete *in situ* closed torso experimental model of ischemia including the generated geometries and electrode locations.

A key requirement for such collections of measurement arrays—often totaling 500–1,000 individual electrodes—is to capture their location in space relative to the anatomy of the heart and torso. To this end, we have developed pipelines consisting of moderate and high-resolution imaging, 3D digitizing, and computational registration tools to create full 3D models with recorded cardiac signals that are customized to each experiment (Rodenhauser et al., 2018; Bergquist et al., 2019a). In brief, we have combined mechanical digitization and gross CT and/or MRI images, obtained either immediately postexperiment or after suitable fixation intervals (several weeks) to identify anatomical structures and electrode locations. A 3D digitizer system (e.g., Microscribe M from GoMeasure3D, Inc, Amherst, VA) is a portable and flexible device that manually captures discrete locations in 3D space, often serving to sample anatomical landmarks and electrode reference points that play a key role in subsequent registration of locations acquired using different modalities and hence difference coordinate frames. We used small-animal computed tomography (CT) to capture cardiac anatomy and needle electrodes from the exercised hearts, after injecting CT contrast agents (e.g., BriteVue, Scarlet Imaging Inc, Murray, UT), and replacing the needles with plastic rods (Burton et al., 2018b; Zenger et al., 2020a). In some cases, we carried out whole-animal MRI scans using clinical 3T scanners (Siemens Medical Solutions USA, Malvern, PA), with MRI-compatible markers used to identify registration points. From the resulting image scans, we segmented organs, electrodes, and registration markers to apply a set of alignment and fitting algorithms and create computer models for each experiment that included electrode locations within the personalized model of each animal (Bergquist et al., 2019a). A final step for experiments we used as the basis for subsequent simulation studies was to convert heart locations into a universal ventricular coordinate system that seeks to normalize anatomical variability into parameterized forms for both the ventricle (Bayer et al., 2018) and atria (Roney et al., 2019; Roney et al., 2021). Figure 3 shows an example of heart and torso geometries that include anatomy and electrode locations for intramural needles and epicardial sock and torso surface arrays.

## 4 Example: Torso-tank preparation

There is a well characterized biophysical relationship between cardiac electrical sources located within the thorax and the ECG’s measured on the torso surface (Gulrajani, 1988; MacLeod et al., 2010), a relationship leveraged qualitatively to interpret clinical ECG measurements (Wagner, 2008). However, there are means to apply quantitative approaches to this scenario, provided the geometric relationship of heart and thorax are known and sufficient ECG signals are available, an approach known as ECG Imaging (ECGI) (Ghanem et al., 2003; Pullan et al., 2010; Cluitmans et al., 2018). ECGI is an imaging modality that seeks to reconstruct cardiac electrical sources from remote ECG signals and as with many imaging approaches, the problem is ill-posed (Gulrajani, 1998) and often requires physical phantoms for validation and refinement (Franzone et al., 1978; Oster et al., 1024; Burnes et al., 2000; Ramanathan and Rudy, 2001; Ghodrati et al., 2006). One such physical phantom is the torso tank preparation, in which a perfused, isolated heart is suspended in an electrolytic tank, often in the shape of a human torso (Burger and van Milaan, 1946; Nagata, 1970; Franzone et al., 1979; MacLeod et al., 1994; Shome and MacLeod, 2007; Bergquist et al., 2021). Such a model requires measurement of high-resolution cardiac and torso potentials with precise geometric information in which one can induce common cardiac pathologies (Ramanathan and Rudy, 2001; Ramanathan et al., 2006; Shome and MacLeod, 2007; Bear et al., 2018; Cluitmans et al., 2018; Cuculich and Robinson, 2018). We describe here an implementation of such a torso-tank preparation, one that represents decades of development and has provided data for hundreds of studies, including for our current focus on acute myocardial ischemia.

### 4.1 Human anatomy and physiology

**FIGURE 4:**
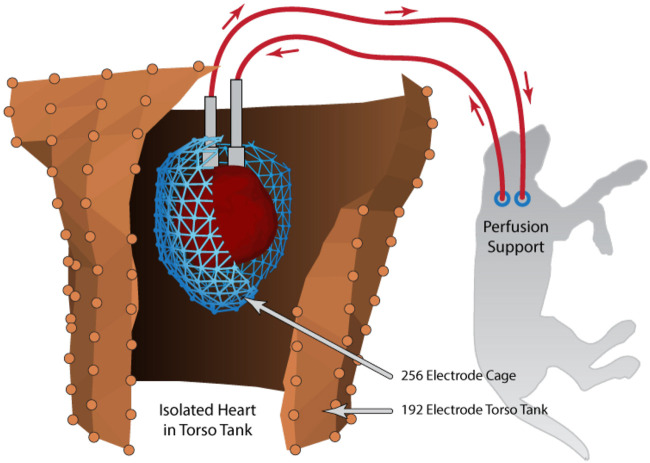
Schematic example of the torso tank preparation that includes an isolated heart, suspended in the human shaped torso tank and perfused by a secondary animal. Recording arrays shown are the pericardiac cage array (blue) surrounding the isolated heart and torso tank array (brown). Reprinted from Bergquist et al. (2021) Copyright 2021, with permission from Elsevier.

The first requirement of this preparation is to simulate the gross anatomy of the human torso within which a functioning heart is positioned. To create a model of suitable scale for a large-mammal heart, we designed a series of torso tanks molded from the same 10-year-old male (Franzone et al., 1978; Ghodrati et al., 2006; Shome and MacLeod, 2007). In each, the heart is suspended within the tank at the equivalent location of the heart within a human torso, and the tank filled with electrolytic fluid approximating the average torso electrical conductivity (Figure 4) (MacLeod et al., 1994; Shome and MacLeod, 2007). The fluid is temperature controlled throughout the experiment to maintain physiological conditions within the tank (Bergquist et al., 2021). The latest in this series of tank designs enables the placement of phantom lungs (or other organs) to mimic torso heterogeneities (MacLeod et al., 1994; MacLeod et al., 1995b).

The isolation and perfusion of the heart are similar to the well-known Langendorff preparation (King et al., 2022), in which the heart is perfused *via* the aorta in the retrograde direction through the coronary arteries. Important modifications for our preparation include the use of isolated canine hearts and providing perfusion support from a second dog (Franzone et al., 1979; Shome and MacLeod, 2007; Bergquist et al., 2021). The blood from the coronaries of the isolated heart exits through the coronary sinus to be collected from the right ventricle and returned to the second, intact animal for oxygenation and maintenance. The use of the support animal and whole blood outperforms other Langendorff preparations that replace blood with artificially oxygenated electrolyte solutions (typically Tyrode’s solution or a mix of blood and Tyrode’s) (White et al., 2015; Bear et al., 2021). The support animal provides a robust physiological filtering system, while typical Langendorff preparations lack the means to clear toxic metabolites accruing throughout the experiment. The second animal also maintains appropriate blood pressure and blood oxygenation without added machinery and the associated complexity. The result has been experiments that have a longer duration (up to 8 h) under stable conditions with limited cardiac swelling, robust mechanical function, and modest accumulation of toxic metabolites. The resulting closed circulatory circuit in our preparation has an additional advantage: limited leakage of blood or perfusate from the heart into the electrolytic fluid in the tank, thus making it easier to maintain electrical conductivity throughout the experiment. While the use of two animals in the isolated-heart preparation is costly, the experiments carried out by our group and others suggest that the data quality and model robustness outperform other, traditional Langendorff-perfused heart models. The net result is a preparation that is more predictable and remains viable longer, producing data of higher quality and quantity, features of special importance when each animal is its own control. The net result is an improvement in both the refinement of the experiments and an overall reduction of such experiments over other models.

The torso tank is vertically oriented and allows flexible positioning of a gantry to which the isolated heart is mounted, thus enabling controlled changes in heart position. Position is particularly relevant in validating ECGI approaches because the heart moves with normal breathing or body position (i.e., lying down or sitting up) (Swenson et al., 2910; Coll-Font and Brooks, 2018). The rigid, calibrated gantry allows controlled, discrete movements with four degrees of freedom and recordings of cardiac and torso potentials at each position (Bergquist et al., 2022a). Heart position is a likely source of error in implementing accurate ECGI, and ongoing studies are focused on evaluating and then correcting the errors that result from position uncertainty (Bergquist et al., 2022a; Bergquist et al., 2022b).

Despite the scientific flexibility of an isolated heart within a torso tank, there are known differences compared to an *in situ* preparation. First, removing the heart from the chest cavity and using retrograde perfusion alters the mechanical function. Retrograde coronary perfusion through the aorta creates a system with no preload on the left ventricle and no afterload–the ventricle is dry—therefore, contraction and metabolic demand are both reduced. We have attempted to simulate preload by placing a fluid-filled and pressurized balloon inside the left ventricular cavity. However, the resultant cardiac signals were similar to experiments without any added preload. A more subtle effect is the absence of intact pericardium in both preparations. Removing the pericardium enables passive cardiac muscle stretch, which could drive changes to contractility *via* responses related to pre-stretch like the Frank-Starling mechanism. Given our focus on electrophysiology, we have not evaluated the effects of such changes on mechanics and considered them likely to be of secondary order compared to the target interventions. A final feature of the isolated-heart preparation is that it is separated from the central nervous system and local hormone regulation. In our experiments, the isolated heart typically showed higher heart rate variability during normal sinus rhythm than observed with *in situ* praprations. We have not found any significant changes to wave propagation, cardiac activation, or repolarization compared to *in situ* experiments.

### 4.2 Pathophysiology

The isolated heart preparation lends itself to a wide range of acute pathologies because it is isolated and perfused, and so has no pumping responsibilities. Thus, it is possible to apply combinations of pacing, acute ischemia, or even, within limits imposed by the support animal, chemical interventions. The major focus of our studies has been the effects of premature ventricular contractions, the electrical response to acute myocardial ischemia, and defibrillation. Acute myocardial ischemia is simulated as described above, with a hydraulic occlusion device around the left anterior descending coronary artery or controlled flow through a cannulated artery, combined with rapid pacing to decrease supply and increase demand, respectively (Shome and MacLeod, 2007; Aras et al., 2011b; Zenger et al., 2020a; Zenger et al., 2020c; Zenger et al., 2021b). The benefits of the support animal are especially critical in the setting of ischemia as it provides metabolic stability and restores electrolyte balance after each ischemic episode, thus allowing multiple repetitions within the same experiment.

The isolated heart also provides direct access to all aspects of the heart, allowing for the placement of intramural needle electrodes and, thus, for comprehensive studies using varied pacing, 3D mapping, and even defibrillation. For the experimental induction of premature ventricular contractions, the intramural recording arrays described above (Zenger et al., 2020a) allow for both comprehensive mapping of the myocardium and flexible pacing from sites within the same region. For example, it is straightforward to initiate PVCs along each electrode pair of each needle and thus stimulate from any depth within the myocardium. At the same time, all the other needle and epicardial electrodes (up to 1,000 in total) are available for capturing the resulting spread of excitation, supporting the exploration of the complex electrical relationships of pacing and myocardial fiber structure (Taccardi et al., 1994; Taccardi et al., 2008; Rupp et al., 2020). It is even possible to use multiple stimulation protocols and sites simultaneously or independently, e.g., to generate validation data to evaluate the precision and resolution of ECGI to locate the earliest sites of activation (Taccardi et al., 1994; Taccardi et al., 2008; Rupp et al., 2020). The same ease of access and the control over perfusion also allow for studies of fibrillation and defibrillation (Tate et al., 2011; Tate et al., 2014).

### 4.3 Measurement system requirements and access

Measurements in this preparation are similar in nature to those in the *in situ* preparation, but benefit from easier access to all aspects of the isolated heart and the comprehensive coverage of the torso-tank surface (384 electrodes). We have implemented similar intramural and sock arrays to those described above; the torso surface electrodes are permanently fixed in the tank (Zenger et al., 2020a). An electrode array that is unique to the tank preparation is a pericardiac cage electrode array, which contains a set of electrodes embedded in a rigid frame that can surround the entire heart and thus is not affected by cardiac movement (Milanic et al., 2014; Tate et al., 2018; Bergquist et al., 2019b; Bergquist et al., 2020; Bergquist et al., 2021). Such cage arrays record signals within millimeters of the cardiac surface, providing highly detailed and complete coverage of cardiac source potentials. The cage is also moveable within the torso tank so that we can study the effects of geometric error. One notable result of all these features is our recent report of a benchmark study for accurate validation measurements of the electrocardiographic forward problem, which forms the core of all ECGI methods (Bergquist et al., 2021). A torso tank can also include up to hundreds of electrodes, both embedded into the surface (in our case, from 384 separate sites) (Shome et al., 2007; Bergquist et al., 2021) and mounted on rods that probe the volume (1,300 in our Utah II torso) (Ni et al., 2000).

**FIGURE 5:**
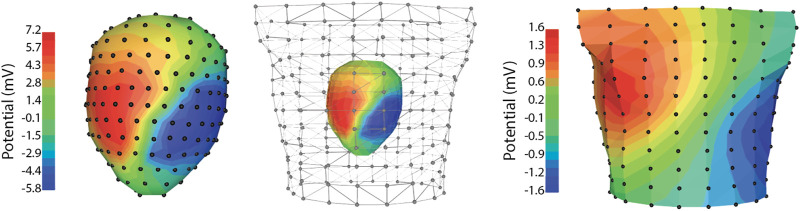
Example data recorded simultaneously from the pericardiac cage array and torso tank of a normal sinus heart beat. Note the significant changes in signal amplitude between the pericardial cage and torso recordings.

Geometric measurements are an essential element of any spatial sampling and they include bringing both the anatomy and the electrode locations into the same spatial frame for analysis. The torso tank preparation facilitates such measurements as so much of the apparatus is rigid and easily accessible. The isolated heart in our preparation is suspended by means of a precisely adjustable gantry system, which can be raised and lowered to both place the heart and provide for careful measurements of its position (Shome and MacLeod, 2007; Bergquist et al., 2021). The shape and electrode locations of the torso tank itself are also well characterized from previous measurements and form a rigid coordinate frame to which all other locations can be referenced, often by means of a mechanical 3D digitizer. To reconstruct the heart anatomy and locations of the intramyocardial needle electrodes, we conduct postmortem imaging, as described above. Figure 5 contains an example of the geometry of the pericardiac cage and torso-surface electrodes with potentials mapped to the surfaces as colors that correspond to voltage amplitude at the time of peak QRS.

## 5 Conclusion

Experimental models of cardiac electrophysiology across all scales are essential to further our understanding of human physiology and pathophysiology. These models provide vital insights into the complex electrical phenomena that occur within the cells and tissues of the heart and how this information projects to the body surface. The whole-body and whole-organ scales are especially advantageous as translational tools to understand and interrogate specific electrical functions because they respond to pathophysiological influences. When developing a whole-body experimental model, it is first important to clearly define the hypothesis and scientific question. From this starting point follow three critical considerations: 1) to understand how the model replicates human physiology, 2) to understand how the model replicates pathophysiology of scientific interest, and 3) to develop measurement systems to detect and record relevant parameters with sufficient spatial coverage and spatiotemporal resolution to answer the scientific question. We have shown through two examples how this framework can easily be applied to the specific study of myocardial ischemia and other questions in cardiac electrophysiology. Applying these principles routinely will increase the chances of success by systematically identifying and selecting all the necessary elements.

## Data Availability

Publicly available datasets were analyzed in this study. This data can be found here: https://edgar.sci.utah.edu.
